# Research progress on the effects and mechanisms of anesthetics on neural stem cells

**DOI:** 10.1002/ibra.12071

**Published:** 2022-11-07

**Authors:** Ji Zhang, Quan‐Yuan Chang, Loris Rizzello, You Wu

**Affiliations:** ^1^ Department of Anesthesiology Southwest Medical University Luzhou China; ^2^ Department of Pharmaceutical Sciences University of Milan Milan Italy; ^3^ National Institute of Molecular Genetics (INGM) Milan Italy; ^4^ Department of Family Planning The Affiliated Hospital of Zunyi Medical University Guizhou Zunyi China

**Keywords:** anesthesia, development, drugs, neural stem cells

## Abstract

Exposure to anesthetic drugs has been proven to seriously affect developing animals in terms of neural stem cells' (NSCs') proliferation, differentiation, and apoptosis. This can severely hamper the development of physiological learning and memory skills. Studies on the effects of anesthetics on NSCs' proliferation and differentiation are thus reviewed here, with the aim to highlight which specific drug mechanisms are the least harmful to NSCs. PubMed has been used as the preferential searching database of relevant literature to identify studies on the effects and mechanisms of NSCs' proliferation and differentiation. It was concluded that propofol and sevoflurane may be the safest options for NSCs during pregnancy and in pediatric clinical procedures, while dexmedetomidine has been found to reduce opioid‐related damage in NSCs. It was also found that the growth environment may impact neurodevelopment even more than narcotic drugs. Nonetheless, the current scientific literature available further highlights how more extensive clinical trials are absolutely required for corroborating the conclusion drawn here.

## Graphical abstract

Numerous studies have shown that exposure of developing animals to anesthetic drugs affects the proliferation, differentiation, apoptosis, and subsequent learning and memory of neural stem cells, which in turn affects learning and memory abilities. We reviewed studies on the effects and mechanisms of four classes of commonly used anesthetics on the proliferation and differentiation of neural stem cells to summarize the drugs that are least clinically harmful for maternal and pediatric intraoperative use. We conclude that propofol and sevoflurane are safer in pregnant and pediatric clinical procedures, but the developmental growth environment may have a greater effect on neurodevelopment than anesthetics.

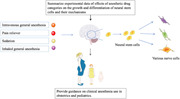

## 1 INTRODUCTION

Neural stem cells (NSCs) are defined as cells having the ability to proliferate, self‐renew, and produce a large number of functional progeny, which can differentiate into neurons, astrocytes, or oligodendrocytes (the three main cell types of the central nervous cell [CNS]). Such ability of NSCs to differentiate into several types of cells, named pluripotency, is finely controlled by genomic, biochemical, and physical factors.^1^, ^2^, ^3^ Studies have shown that gene expressions, as well as the ability of NSCs to self‐renew and differentiate, are spatiotemporally specific. NSCs are available throughout the development process and continue to exist in the adults' nervous systems.^4^

Although NSCs have some well‐defined characteristics, the environment in which they are exposed can alter their fate. Most of the general anesthetics currently in use have *N*‐methyl‐d‐aspartic (NMDA)‐type glutamate receptor blocking or γ‐aminobutyric acid (GABA) receptor‐enhancing properties.^5^ In vivo and in vitro experimental data show that exposure of developing animals to anesthetics would lead to harmful effects on NSCs' proliferation, differentiation, apoptosis, and later on learning and memory skills.^4^ The effects of anesthetics on NSCs are related to the following factors: (1) the type of the anesthetic, (2) the dosage, (3) the duration or interval of exposure, (4) the combined use of other drugs, and (5) the age and the physiological condition of animals.^6^ It is reported that there is also an association between general anesthesia exposure in infancy and neurobehavioral problems in childhood for humans.^7^ Understanding the potential neurotoxicity of anesthetic is thus of utmost importance, especially in the field of pediatric anesthesiology.^7^, ^8^, ^9^, ^10^, ^11^, ^12^ This is particularly true because of the significant increase in the need for fetal sedation and analgesic interventions, and for the concerns about fetal‐related anesthetic neurotoxicity, upon maternal anesthesia.

Current reviews of studies on the toxicity of anesthetic drugs on NSCs were published too early to describe the mechanisms of drug action in detail. The effects of anesthetic drug categories on the growth of NSCs and their mechanisms from drug categories were thus reviewed in this study. References for maternal and pediatric clinical operations and stimulation of the development of new anesthetics based on experimental data are also provided.

## 2 EXPERIMENTAL MODELS AND EXPERIMENTAL METHODS

**Figure 1:**
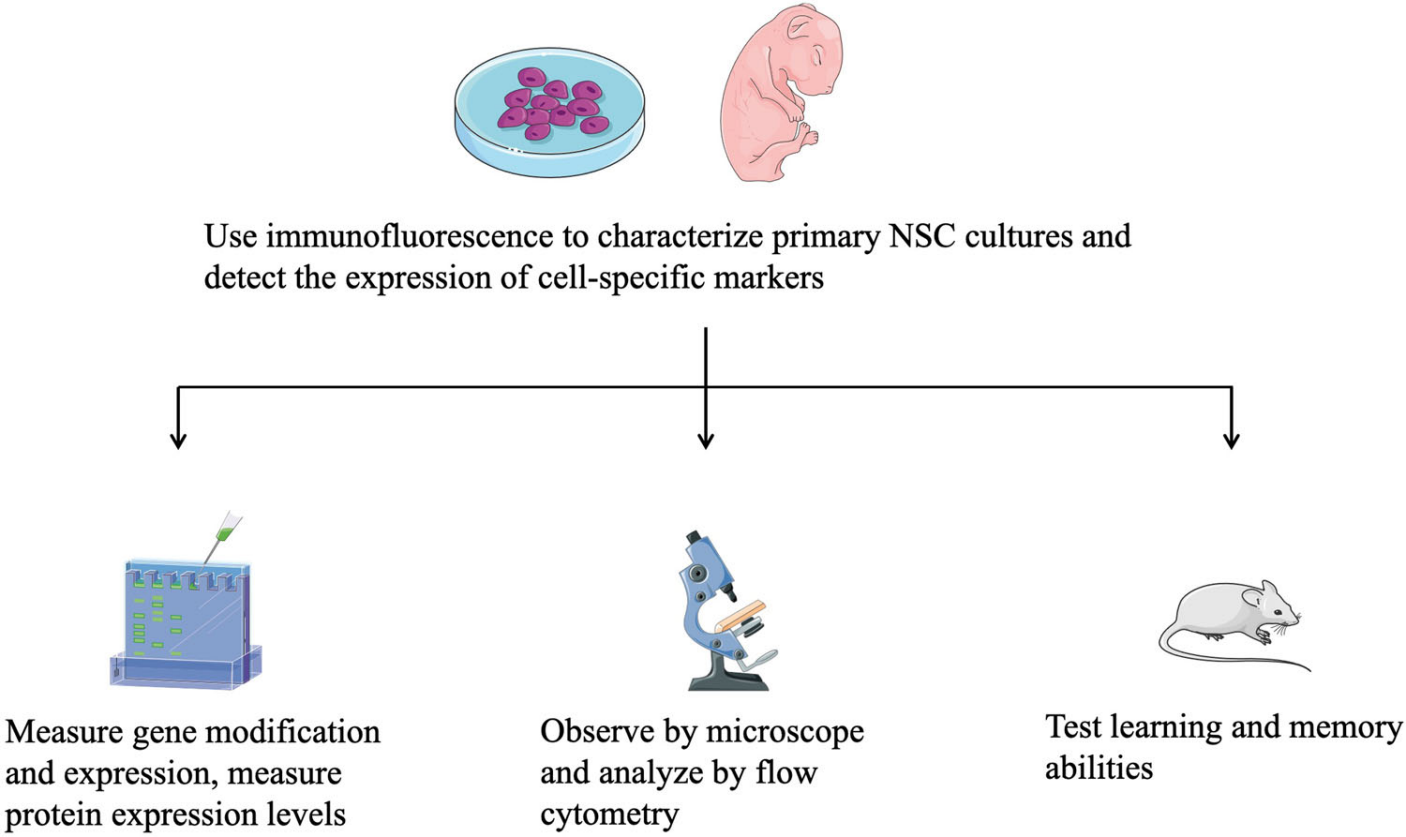
Main experimental procedures were as follows: Characterize primary neural stem cell (NSC) cultures using immunofluorescence to ensure successful modeling; detect the expression of cell‐specific markers of NPs to confirm cell type; use bioinformatics analysis to find possible target genes or pathways and then measure gene modification and expression in experiments; measure protein expression levels to assess the living state of cells; ion concentrations were analyzed by flow cytometry; cell growth morphology, membrane integrity, and cell counts were performed by microscopy to assess the differentiation, proliferation, and cell cycle kinetics of NSCs; behavior, learning, and memory abilities were determined using the Morris water maze, buried food test, and olfactory memory test. This figure was modified from Servier Medical Art (http://smart.servier.com/), licensed under a Creative Common Attribution 3.0 Generic License (https://creativecommons.org/licenses/by/3.0/). [Color figure can be viewed at wileyonlinelibrary.com]

Mice, rats, or human stem cells are often used as models to study the effects of different anesthetics on NSCs' development. Most experiments were in vitro studies using human and murine stem cells. There are only a few in vivo studies based on mice or rat animal models. Therefore, before the conclusions could be applied in clinical practice, they need the support of further clinical experimental data. The value of the conclusions is to provide guidance for further research. Representative procedures and experimental methods are as follows^13^, ^14^, ^15^, ^16^, ^17^, ^18^, ^19^, ^20^, ^21^, ^22^, ^23^ (Figure 1 and Table 1).

**Table 1:** Main studies on the effects of drugs on neural stem cells and their mechanisms

Classification	Anesthetic	Receptor	Model	In vivo	Dose	Pathway	Effect	References
Intravenous general anesthesia	Ketamine	NMDAR	Human NSCs	−	0–100 μM	PI3K/Akt–p27 signaling, Ca(2+)–PKCα–ERK1/2 signaling pathway, Notch1 signaling pathway	Learning and memory impairment	[13, 15, 24]
Pain reliever	Morphine	Opioid receptors	Cerebral cortex of day 14 ICR mouse embryos	−	0, 0.13, 1.3, and 13 µM	Increased expression of caspase‐3	Inhibit proliferation	[16]
	Oxycodone	Opioid receptors	D14 rat embryo	−	0.1, 1.0, and 10 μg/ml	−	Helps brain development	[17]
Sedative	Propofol	NMDAR and GABAR	Cortical and hippocampal NSCs of E16–E18 SD rat embryos	−	50 μM	miR‐124‐3p/Sp1/cdkn1b axis	Promote differentiation	[18]
	Midazolam	GABAR	Intraperitoneal injection of P7 mice	+	10 mg/kg	Calcium influx, changes chromatin	Proliferation is restricted and memory function is impaired	[19]
	Diazepam	GABAR	ICR (CD1) mice	+	30 mg/kg	−	Blocked NSC proliferation induced by MCAO	[20]
	Dexmedetomidine	α‐2‐AR	E18 SD rat embryonic precortex	−	0.1, 1.0, 10, and 20 µM	PI3K/Akt/GSK‐3β signaling pathway	Increase proliferation, decrease apoptosis	[21]
Inhaled general anesthesia	Isoflurane	GABAR	E13 mouse striatal NSC	−	0, 0.125, 0.25, and 0.5 mM	Lkb1–p53–p21 signaling pathway	Inhibit proliferation	[22]
	Sevoflurane	GABAR	Pregnant rats, P7 mice	−	2%, 3.5%	Wnt/β‐catenin signaling pathway	Low‐dose, short‐term effect is not obvious	[25]

*Note*: In the fifth column, “+” is for in vivo and “−” is for in vitro. In the seventh column, “−” is for unknown.

Abbreviations: α‐2‐AR, α‐2 adrenergic receptor; GABAR, γ‐aminobutyric acid receptor; MCAO, middle cerebral artery occlusion; NMDAR, *N*‐methyl‐d‐aspartate; NSC, neural stem cell; SD, Sprague–Dawley.

## 3 EFFECTS OF ANESTHETIC DRUGS ON NSCs AND THEIR MECHANISMS

Anesthetics have analgesic and sedative effects by reversibly blocking nerve conduction. However, the anesthetic can be toxic to NSCs by acting on different receptor‐mediated signaling pathways and thus affecting NCS growth, proliferation, and differentiation.

### 3.1 Intravenous general anesthetics, sedatives, and analgesics

Intravenously administered anesthetics are drugs acting on the CNS through blood circulation and are meant to produce general anesthesia. Some examples include ketamine, morphine, oxycodone, propofol, midazolam (MDZ), diazepam, and dexmedetomidine (DEX) (Table 1).

#### 3.1.1 Ketamine

Ketamine is a short‐acting anesthetic that can be administered alone. It can also be used as an inducer of other general anesthesia and auxiliary anesthesia, with weaker effects, but it can be used in combination with other general or local anesthesia. Studies performed in neurodissociation model rats have shown that exposure to ketamine inhibits hippocampal NSC proliferation and differentiation, reduces astroglial differentiation, and causes neuronal apoptosis, interfering with hippocampal neurogenesis and long‐term neurocognitive function.^24^

**Figure 2:**
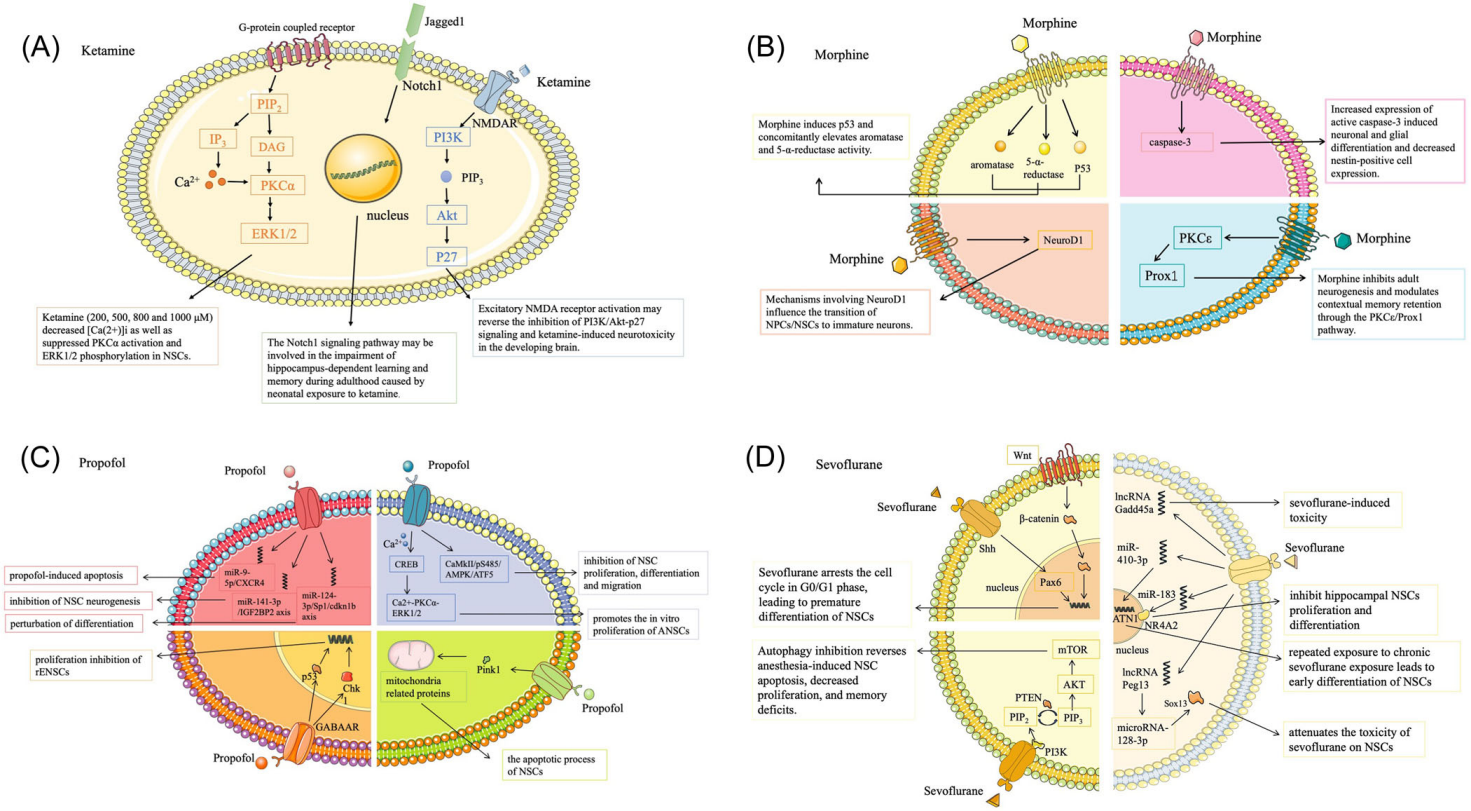
Diagram of the mechanism of action of ketamine, morphine, propofol, and sevoflurane on neural stem cells. (A) Notch1 signaling is associated with neonatal ketamine exposure‐induced impairment of hippocampus‐dependent learning and memory in adulthood. The Ca^2+^‐PKCα‐ERK1/2 signaling pathway may be involved in ketamine inhibition of proliferation of NSCs. However, activation of excitatory NMDAR may reverse the inhibition and ketamine‐induce neurotoxicity in the developing brain. (B) Morphine induces p53 and increases both aromatase and 5‐a‐reductase activity, and increased expression of active caspase‐3 induces neuronal and glial differentiation. The mechanism of morphine involving NeuroD1 affects the conversion of NPCs/NSCs to immature neurons. Morphine also regulates retention and decline of situational memory via the PKCε/Prox1 pathway. (C) Propofol inhibition of neurogenesis in NSCs was associated with the miR‐141‐3p /IGF2BP2 axis, whereas perturbation of differentiation was associated with the miR‐124‐3p/Sp1/cdkn1b axis. Propofol inhibits the proliferation, differentiation and migration of NSCs through the CaMkII/pS485/AMPK/ATF5 signaling pathway. The Ca^2+^‐mediated pathway promotes the proliferation of adult NSCs in vitro. Exogenous and endogenous apoptotic pathways induce apoptosis in stem cells, and GABAA (ion receptor for γ‐aminobutyric acid) receptor‐mediated inhibition of apoptosis and proliferation in rat embryonic neural stem cells (rENSCs) may be regulated by the Chk1/p53 signaling pathway. In addition, miR‐9‐5p/CXCR4 contributes to propofol‐induced apoptosis, Pink1‐mediated mitochondrial pathway and signal transducer and transcriptional activator 3/miR‐21/Sprouty2‐dependent mechanisms. Thus propofol plays an important role in the apoptosis of NSCs. (D) Sevoflurane acts on lncRNA Gadd45a to inhibit the proliferation and differentiation of hippocampal NSCs by interacting with miR‐183 and Nuclear Receptor Subfamily 4, Group A Member 2 (NR4A2) and c‐Jun N‐terminal kinase (JNK) pathways. However, the long non‐coding RNA Peg13 attenuated the toxicity of sevoflurane to NSCs by sparing microRNA‐128‐3p to maintain the expression of Sox13. Sevoflurane was associated with decreased levels of epidermal growth factor (EGF), phosphoinositide‐3‐kinase (PI3K) and Phospho‐AKT proteins in second‐quarter NSCs apoptosis. Sevoflurane halted the cell cycle at G0/G1 phase by inhibiting the wnt/β‐catenin signaling pathway, which led to premature differentiation of NSCs. And the inhibition of neurogenesis by activating the Pax6 pathway has deleterious effects on fetal and offspring brain development. More, maternal exposure to sevoflurane during peak neurogenesis affects NSCs in the fetal brain via the Notch signaling pathway. sevoflurane also induces excessive autophagy in the fetal brain via the PTEN/Akt/mTOR pathway [Color figure can be viewed at wileyonlinelibrary.com]

Ketamine‐induced NSC differentiation toxicity is associated with functional N‐methyl‐d‐aspartic acid receptor (NMDAR). Excitatory NMDAR activation may reverse the inhibition of PI3K/Akt‐p27 signaling and ketamine‐induced neurotoxicity in developing brains.^13^, ^26^ Ketamine also hinders neuroprotection associated with Lin28b upregulation.^27^ The Ca^2+^–PKCα–ERK1/2 signaling pathway may be involved in the inhibition of NSCs' proliferation.^15^ Notch1 signaling has been implicated in neonatal ketamine exposure‐induced impairment of hippocampal‐dependent learning and memory in adulthood^24^ (Figure 2A). Some drugs can reduce the damage caused by ketamine and provide protection to NSCs (Table 2).

**Table 2:** Effects of other drug effects on ketamine neural stem cell toxicity

Drug	Research object	Model	In vivo	Pathway	Protect	References
Clozapine	Lateral ventricle SVZ	6‐week‐old adult male mice	−	Caspase‐3	Yes	[28]
Dexmedetomidine	Brain	PND‐7 male rat	+	Ca ^2+^–PKCα–ERK1/2 signaling pathway	Yes	[29]
17β‐estradiol	Junior NSPC	Sprague–Dawley rats at E18 and E19	−	ER‐β, GSK‐3β pathway participate	Yes	[30]
17β‐estradiol	Brain tissues in SVZ and SGZ	PND‐7 rat	+	Spatial learning and memory decline in adulthood	Yes	[31]
PACAP	Lateral ventricle SVZ	6‐week‐old adult male mice	−	mTOR signaling pathway	Yes	[32]
Minocycline	Cerebral cortex	Sprague–Dawley rats at E18 and E19	+	PI3K/Akt/Gsk‐3β signaling pathway	Yes	[33]

*Note*: “+” is for in vivo; “−” is for in vitro.

Abbreviations: NSCP, neural stem/progenitor cell; PACP, pituitary adenylate cyclase‐activating polypeptide; PI3K, phosphoinositide‐3‐kinase; PND, postnatal day; SGZ, subgranular zone; SVZ, subventricular zone.

#### 3.1.2 Morphine

Morphine has a powerful analgesic effect and it is effective in the treatment of all severe pains. It is more effective for persistent dull pains than intermittent sharp pains and visceral pains. Exposure to morphine sulfate (MS) at the early and late stages of cell differentiation significantly reduces the genotype and phenotype of differentiated neuronal cells.^34^ Experiments have also shown that morphine induces the upregulation of p53, and it also promotes the activity of aromatase and 5‐α‐reductase. It thus severely impairs the health of NSCs in the early 48 h, hindering the possibility to trigger angiogenesis.^35^ Morphine has also been proven to inhibit rats' NSCs' proliferation rate by increasing neurosteroidogenesis. It also boosts the expression of active caspase‐3‐induced neuronal and glial differentiation and decreases nestin‐positive cell expression. Naloxone is used to time dependently attenuate the enhanced neurosteroidogenesis induced by morphine.^36^ In vivo, morphine prevents NSCs' proliferation associated with decreased testosterone levels, increased dihydrotestosterone levels, and p53 overexpression.^37^

The effects of morphine on normal differentiation of NSCs are related to changes in p300, H3K27ac, DNA methylation, and Oct4, Sox2, and Nanog to alter nestin.^38^ Irregularity effects of morphine on NSC kinetics and activity are associated with reduced insulin and insulin‐like growth factor secretion and downregulation of insulin receptors.^39^ Mechanisms involving NeuroD1 influence the transition of neural progenitor/stem cells toward immature neurons under the conditional place preference experiment training paradigm.^40^ Morphine mediates spectrum‐specific neural stem/progenitor cell (NSPC) differentiation, inhibits adult neurogenesis, and regulates contextual memory retention and decline via the PKCε/Prox1 pathway^41^ (Figure 2B).

#### 3.1.3 Oxycodone

Oxycodone is used in children and adults to treat acute postoperative pain. Oxycodone takes effect by binding to mu receptors in CNS. Such interaction mimics the effects of endogenous opioids and prevents the production of cyclic adenosine monophosphate (cAMP) followed by the release of nociceptive neurotransmitters. Furthermore, oxycodone closes N‐type voltage‐gated calcium channels and opens G‐protein‐coupled inward‐rectifying potassium channels, resulting in hyperpolarization and decreased neuronal excitability (https://pubchem.ncbi.nlm.nih.gov/compound/oxycodone). NSCs' survival rate and proliferation were significantly reduced upon exposure to a high dose of oxycodone (10 g/ml) for 48 h, while NSCs' apoptosis and differentiation were enhanced.^17^ Related experiments found that short‐term exposure to low doses of oxycodone may contribute to brain development.^17^

#### 3.1.4 Propofol

Propofol is a short‐acting intravenous anesthetic that can be used for the induction and maintenance of general anesthesia in surgery. While clinically relevant doses of propofol have no significant effect on the proliferation of rats' NSCs cultured in vitro, they can induce NCS differentiation into neuron‐like cells.^42^ On the other side, high doses of propofol inhibit proliferation, differentiation, and migration of NSCs.^43^ High propofol doses can also induce stem cells' apoptosis, impair cells' proliferation, and inhibit neurogenesis in immature mice' brains, possibly due to its induction of cognitive dysfunction.^44^, ^45^

Studies have shown that acutely and chronically dysregulated miRNA–mRNA signaling networks may be involved in propofol‐induced developmental neurotoxicity.^46^ Two miRNA clusters (Gabbr1 and Cacna1b and Gabbr2), which exhibited differential expression upon exposure, may simultaneously regulate multiple genes in a coordinated manner during NSC development.^47^ Inhibition of NSCs' neurogenesis is associated with the miR‐141‐3p/IGF2BP2 axis,^48^ while perturbation of differentiation is related to the miR‐124‐3p/Sp1/cdkn1b axis.^18^ However, the mechanism of propofol's action on miRNA and its target genes remains to be elucidated, especially on neurons.^42^ Propofol can enhance cAMP‐response‐element‐binding protein (CREB) phosphorylation and activate Ca^2+^–PKCα–ERK1/2 through CaMkII/pS485/AMPK/ATF5 signaling pathway‐mediated inhibition of NSCs' proliferation, differentiation, and migration.^43^ The Ca^2+^‐mediated pathway thus promotes the in vitro proliferation of adult NSCs.^14^, ^49^ Stem cells' apoptosis induced from exogenous and endogenous apoptotic pathways,^50^ GABAA (ionotropic receptors for γ‐aminobutyric acid) receptor‐mediated apoptosis, and proliferation inhibition of rat embryonic NSCs may be regulated by the Chk1/p53 signaling pathway.^51^ Also, miR‐9‐5p/CXCR4 contributes to propofol‐induced apoptosis,^52^ Pink1‐mediated mitochondrial pathways, and both signal transducers and activators of transcription 3/miR‐21/Sprouty2‐dependent mechanisms. Propofol plays an important role in NSCs' apoptosis.^53^ Together with remifentanil, they interfere with the proliferation and differentiation of neural stem/progenitor cells by altering [Ca(2+)] ions. However, the neurotoxicity can be mitigated by monosialicacidganglioside 1^54^, ^55^ (Figure 2C).

#### 3.1.5 MDZ

MDZ is a benzodiazepine drug, which acts through GABA neurotransmitters. MDZ causes drowsiness and it is not conducive to developing new memories. Early exposure to MDZ persistently alters chromatin and the expression of quiescence‐related genes in mice's hippocampal NSCs. This results in persistently restricted NSCs' proliferation till adulthood, reduced neurogenesis, and impaired hippocampal‐dependent memory function.^19^ The MDZ‐triggered calcium influx within NSCs can be partly attributed to the activation of GABAA, possibly triggering an inhibitory effect on NCSs' proliferation.^56^ Minocycline pretreatment can reduce the damage of MDZ on the proliferation of NSCs in neonatal rats and improve the spatial learning and memory ability of adult rats.^57^

#### 3.1.6 Diazepam

Diazepam is a benzodiazepine derivative that enhances the inhibitory activity of GABA by binding to GABA receptors located in the limbic system and hypothalamus. This increases the frequency of chloride channel opening, allowing the flow of chloride ions into the neuron and ultimately leading to membrane hyperpolarization and a decrease in neuronal excitability. It thus possesses anxiolytic, sedative, hypnotic, and anticonvulsant properties (https://pubchem.ncbi.nlm.nih.gov/compound/diazepam). Long‐term use can be addictive, and large doses may have side effects such as ataxia, tremor, anterograde amnesia, and cognitive impairment.^58^ Diazepines are neurotoxic to hippocampal cells, cause abnormal behavioral findings, and are associated with an increased risk of dementia.^59^ It could elevate hippocampal activity and NSCs' proliferation after middle cerebral artery occlusion.^20^

#### 3.1.7 DEX

The main pharmacological component of DEX is the α2‐adrenergic receptor antagonist, which binds to the corresponding receptors in the human body, thereby inhibiting the excitatory state of sympathetic nerves. DEX has been suggested to prevent inhalation anesthetic‐induced neurotoxicity in the developing brain, using neonatal animal models of anesthesia.^60^ DEX may protect NSCs from ketamine‐induced damage through the PI3K/Akt/GSK‐3β signaling pathway.^21^ Pretreatment with moderate to high (5 μg/kg) or high dose (10 μg/kg) of DEX for 24 h, after repeated ketamine exposure in the neonatal period, disrupted ketamine‐induced proliferation and differentiation of NSCs in the development of the subventricular zone with neuroprotective effect.^29^ DEX cotreatment attenuated MDZ‐induced changes in cells' proliferation, viability, apoptosis, and protein expression of p‐c‐Jun N‐terminal kinase (JNK) and p‐P38 in cultured NSCs. However, MDZ and DEX both have not affected the differentiation of cultured NSCs.^61^

### 3.2 Inhaled general anesthesia

Inhalation anesthetics are inhaled through the lungs. They pass the alveolar–capillary membrane and diffuse into the blood, ultimately reaching the CNS and inducing anesthesia.^62^ The efficacy of inhaled anesthetics depends on the minimum alveolar concentration (MAC). There are many studies related to isoflurane and sevoflurane, so these two inhalation anesthetics are mainly introduced (Table 1).

#### 3.2.1 Isoflurane

Isoflurane inhibits the conduction of opened potassium channels. It can also activate the glutamate receptors, glycine receptors, and GABA receptors, which leads to the inhibition of motor function as a final outcome.^63^ Isoflurane does not kill NSCs in vitro but inhibited the proliferation of these cells at, or above, the MAC required for general anesthesia (1.4% and 2.8%), but not below 0.7%.^64^ Isoflurane does not cause cell death, but it acts directly on neural progenitor cells by reducing proliferation and increasing neuronal fate selection. Activation of the Lkb1–p53–p21 signaling pathway inhibits self‐renewal of NSCs in mice. These changes may have detrimental effects on cognition after isoflurane anesthesia,^22^, ^65^ the magnitude of which is clearly age‐dependent.^66^ The JNK inhibitor alleviates emulsified isoflurane‐induced apoptosis of fetal NSCs.^67^ l‐Theanine and simvastatin attenuate isoflurane‐induced neurogenetic damage and neurocognitive deficits in developing rats' brains through upregulation of the Akt/GSK‐3β signaling pathway.^68^, ^69^

#### 3.2.2 Sevoflurane

Sevoflurane is a halogenated ether anesthetic that induces and maintains general anesthesia. The concentration of sevoflurane can produce certain cytotoxicity by affecting the methylation degree of NSCs in rats.^23^ Repeated exposure to chronic sevoflurane leads to early differentiation of NSCs through the miR‐410‐3p/ATN1 pathway,^70^ reducing their pluripotency, reserves, and hypoxia tolerance.^71^ Sevoflurane inhibits the CNS by activating GABAA, leading to apoptosis and degeneration of NSCs.^72^ However, 2 h treatment with sevoflurane did not produce significant changes in the survival, proliferation, apoptosis, and differentiation of human NSCs.^73^ Nonetheless, prolonged exposure to sevoflurane may reduce the self‐renewal capacity of hippocampal NSCs, leading to cognitive deficits.^74^ Sevoflurane‐mediated endoplasmic reticulum stress has differential effects on different subregions of the immature hippocampus. Inhibition of endoplasmic reticulum stress would be, in turn, a potential approach to prevent subsequent learning and memory deficits in adulthood.^75^ Maternal exposure to high concentrations of sevoflurane (3.5%) during pregnancy can induce apoptosis of NSCs, resulting in abnormal neurodevelopment, learning, and memory dysfunction in offspring.^76^ Neurocognitive abnormalities induced by repeated exposure to sevoflurane are exacerbated by stressful conditions such as social isolation and enrichment of deprivation (adverse environment).^74^, ^77^

Experiments have shown that lncRNA Gadd45a is associated with sevoflurane‐induced toxicity.^78^ A potential novel mechanism is to inhibit hippocampal NSCs' proliferation and differentiation by interacting with miR‐183 and nuclear receptor subfamily 4, group A member 2, and the JNK pathway.^79^, ^80^ It is associated with decreased levels of the epidermal growth factor, phosphoinositide‐3‐kinase (PI3K) and phospho‐AKT proteins in NSCs' apoptosis during the second trimester.^76^ Sevoflurane stops the cell cycle in the G0/G1 phase by inhibiting the Wnt/β‐catenin signaling pathway. This results in premature differentiation of NSCs, inhibition of fetal NSCs' proliferation in a dose‐dependent manner, and impair of postnatal learning and memory function.^25^, ^81^ Inhibition of neurogenesis by the Pax6 pathway has deleterious effects on fetal and offspring brains' development.^82^ Maternal exposure to sevoflurane during peak neurogenesis affects fetal brains' NSCs via the Notch signaling pathways but has no long‐term effects on neurocognitive outcomes^83^ (Figure 2D).

Long noncoding RNA Peg13 attenuates the toxicity of sevoflurane on NSCs by sponging microRNA‐128‐3p to maintain Sox13 expression.^84^ A 2‐h exposure to 3.5% sevoflurane at G14 (gestational day 14) induces hyperautophagy in the fetal brain via the PTEN/Akt/mTOR pathway. Autophagy inhibition reverses anesthesia‐induced NSCs' apoptosis, decreased proliferation, and memory deficits.^85^

## 4 CONCLUSION AND PROSPECT

Anesthetics have been proven to have different effects on NCSs (Table 3). While most drugs have negative effects on neurodevelopment, DEX and oxycodone have instead protective effects on the nervous system under certain conditions. We concluded that propofol and sevoflurane have protective effects on NSCs during pregnancy and pediatric clinical surgery and DEX attenuates the damaging effects of opioids on NSCs. Short‐term use of sevoflurane has no significant effect. In the case of long‐term exposure to sevoflurane, anesthetics neurotoxicity together with the influence of growth and development environment should be considered to avoid exacerbation of neurocognitive abnormalities. This is due to stressful conditions such as social isolation and harsh environments. As for short‐term exposure, the growth environment during development may have a greater impact on neurodevelopment than anesthetics.

**Table 3:** Summary of the effects of different anesthetic drugs on neural stem cells

Classification	Anesthetic	Stemness maintenance	Proliferation	Differentiation	References
Intravenous general anesthesia	Ketamine	↓	↓	↓	[6]
Pain reliever	Morphine	↓	↓	−	[16, 36, 86]
	Oxycodone	↓	↓	↓	[17]
Sedative	Propofol	↓	−	↑	[18]
	Midazolam	↓	↓	−	[19, 20, 56]
	Diazepam	↓	↓	−	[20]
	Dexmedetomidev(attenuates ketamine neurogenesis damage)	↑	↑	↑	[21, 61]
Inhaled general anesthesia	Isoflurane	↓	↓	↑	[64]
	Sevoflurane (higher clinical doses)	↓	−	↓	[73]

*Note*: “↑” is for up, “↓” is for down, and “−” is for unknown or no significant change.

Most of the experiments cited in this review are the results of in vitro culturing of rodent or human embryonic stem cell‐derived NSCs, which are different from the environment in which NSCs develop in humans. Therefore, the value of the conclusion is limited. Follow‐up studies will require more in vivo experiments to further investigate the effects of anesthetics on NSC development and lay the foundation for future clinical trials.

## AUTHOR CONTRIBUTIONS

Ji Zhang developed the idea for the study and wrote the paper. Quan‐Yuan Chang helped in writing the paper. Loris Rizzello and You Wu participated in the revision of the article. All authors contributed to the writing and revisions.

## CONFLICT OF INTEREST

Loris Rizzello is a member of the *Ibrain* Journal editorial board and is not involved in the peer review process of this article. The remaining authors declare no conflict of interest.

## ETHICS STATEMENT

Not available. Consent for publication was obtained from all participants.

## Data Availability

Data sharing is not applicable to this article as no new data were created or analyzed in this study.
